# Supplementary material for: Evolutionary adaptation after crippling cell polarization follows reproducible trajectories
Source: eLife. 2015 Oct 1;4:e09638. doi: 10.7554/eLife.09638 (PMC4630673; doi:10.7554/eLife.09638)
Supplement: Supplementary file 2. — Yeast strains. DOI: http://dx.doi.org/10.7554/eLife.09638.019 [file elife09638s002.docx]

**Laan et al, Supplementary file 2**

| **­­Strain name** | **Genetic change** | **Additional Information** |
| --- | --- | --- |
| yLL03a | *MATalpha , can1-100, leu2-3,112, his3-11,15, ura3∆, BUD4-w303* | Ancestor , used as starting point |
| yLL57b | *CAN1/ P_mfa_ HIS3@can1 [1], leu2-3,112/leu2-3,112, his3-11,15/his3-11,15, ura3∆/ura3∆, BUD4-w303/BUD4-w303, BEM1/bem1::KanMX6* | Ancestor diploid that was sporulated to acquire the two *bem1∆* lines (T2/3) used in the pilot evolution experiment |
| yLL66a | *CAN1/ P_mfa_ HIS3@can1, leu2-3,112/leu2-3,112, his3-11,15/his3-11,15, ura3∆/ura3∆, BUD4-w303/BUD4-w303, BEM1/bem1::KanMX6, ABP140/ABP140-mCherry-NATMX4* | Ancestor diploid that was sporulated to acquire the 9 *bem1∆* lines (A1, A2, A4-A10 ) |
| yLL112a | *P_mfa_ HIS3@can1 / P_mfa_ HIS3@can1, leu2-3,112/leu2-3,112, his3-11,15/his3-11,15 ura3∆/ura3∆, BUD4-w303/BUD4-w303, BEM1/bem1::KanMX6, BEM3/bem3::NATMX4, NRP1/nrp1::HPHMX4, SPA2_Citrine_URA3/SPA2* | Diploid used for reconstruction of various mutant haploid strains. |
| yLL135a | *P_mfa_ HIS3@can1 / P_mfa_ HIS3@can1, leu2-3,112/leu2-3,112, his3-11,15/his3-11,15 ura3∆/ura3∆, BUD4-w303/BUD4-w303, BEM1/bem1::KanMX6, BEM2/bem2::LEU2, BEM3/bem3::NATMX4, NRP1/nrp1::HPHMX4, SPA2_Citrine_URA3/SPA2* | Diploid used for reconstruction of various mutant haploid strains as well as for the spore growth analysis in Figure 3B. |
| yLL128a | *MATa, P_mfa_ HIS3@can1, leu2-3,112, his3-11,15, ura3∆, BUD4-w303, bem1::KanMX6, SPA2_Citrine_URA3* | *bem1∆* reconstructed strain |
| yLL129a | *MATa, P_mfa_ HIS3@can1, leu2-3,112, his3-11,15, ura3∆, BUD4-w303, bem1::KanMX6, bem3::NATMX4, SPA2_Citrine_URA3* | *bem1∆bem3∆* reconstructed strain |
| yLL130a | *MATa, P_mfa_ HIS3@can1, leu2-3,112, his3-11,15, ura3∆, BUD4-w303, bem1::KanMX6, nrp1::HPHMX4, SPA2_Citrine_URA3* | *bem1∆nrp1∆* reconstructed strain |
| yLL145a | *MATa, P_mfa_ HIS3@can1, leu2-3,112, his3-11,15, ura3∆, BUD4-w303, bem1::KanMX6, bem2::LEU2, SPA2_Citrine_URA3* | *bem1∆bem2∆* reconstructed strain |
| yLL131a | *MATa, P_mfa_ HIS3@can1, leu2-3,112, his3-11,15, ura3∆, BUD4-w303, bem1::KanMX6, bem3::NATMX4 , nrp1::HPHMX4, SPA2_Citrine_URA3* | *bem1∆bem3∆nrp1∆* reconstructed strain |
| yLL146a | *MATa, P_mfa_ HIS3@can1, leu2-3,112, his3-11,15, ura3∆, BUD4-w303, bem1::KanMX6, bem2::LEU2, bem3::NATMX4, SPA2_Citrine_URA3* | *bem1∆bem2∆ bem3∆* reconstructed strain |
| yLL147a | *MATa, P_mfa_ HIS3@can1, leu2-3,112, his3-11,15/his3-11,15, ura3∆, BUD4-w303, bem1::KanMX6, bem2::LEU2, nrp1::HPHMX4, SPA2_Citrine_URA3* | *bem1∆bem2∆nrp1∆* reconstructed strain |
| yLL148a | *MATa, P_mfa_ HIS3@can1, leu2-3,112, his3-11,15, ura3∆, BUD4-w303, bem1::KanMX6, bem2::LEU2, bem3::NATMX4, nrp1::HPHMX4, SPA2_Citrine_URA3* | *bem1∆bem2∆ bem3∆nrp1∆* reconstructed strain |
| yLL132a | *MATa, P_mfa_ HIS3@can1, leu2-3,112, his3-11,15, ura3∆, BUD4-w303, SPA2_Citrine_URA3* | Wild type reconstructed strain |
| yLL138a | *MATa, P_mfa_ HIS3@can1, leu2-3,112, his3-11,15, ura3∆, BUD4-w303, bem3::NATMX4 , SPA2_Citrine_URA3* | *bem3∆* reconstructed strain |
| yLL137a | *MATa, P_mfa_ HIS3@can1, leu2-3,112, his3-11,15, ura3∆, BUD4-w303, nrp1::HPHMX4, SPA2_ Citrine _URA3* | *nrp1∆* reconstructed strain |
| yLL140a | *MATa, P_mfa_ HIS3@can1, leu2-3,112, his3-11,15, ura3∆, BUD4-w303, bem2::LEU2, SPA2_ Citrine_URA3* | *bem2∆* reconstructed strain |
| yLL143a | *MATa, P_mfa_ HIS3@can1, leu2-3,112, his3-11,15, ura3∆, BUD4-w303, bem3::NATMX4, nrp1::HPHMX4, SPA2_ Citrine_URA3* | *bem3∆nrp1∆* reconstructed strain |
| yLL141a | *MATa, P_mfa_ HIS3@can1, leu2-3,112, his3-11,15, ura3∆, BUD4-w303, bem2::LEU2, bem3::NATMX4, SPA2_ Citrine_URA3* | *bem2∆ bem3∆* reconstructed strain |
| yLL142a | *MATa, P_mfa_ HIS3@can1, leu2-3,112, his3-11,15, ura3∆, BUD4-w303, bem2::LEU2, nrp1::HPHMX4, SPA2_ Citrine_URA3* | *bem2∆ nrp1∆* reconstructed strain |
| yLL144a | *MATa, P_mfa_ HIS3@can1, leu2-3,112, his3-11,15, ura3∆, BUD4-w303, bem3::NATMX4, bem2::LEU2, nrp1::HPHMX4, SPA2_ Citrine_URA3* | *bem2∆ bem3∆nrp1∆* reconstructed strain |
| Evolved yeast strains |  |  |
| T2_X/ T3_X | *MATa, P_mfa_ HIS3@can1, leu2-3,112, his3-11,15, ura3∆, BUD4-w303, bem1::KanMX6* | Evolved haploids that grew from spores from yLL57b (X indicates the number of evolved generations) |
| A1_X, A2_X, A4_X-A10_X | *MATa, P_mfa_ HIS3@can1, leu2-3,112, his3-11,15, ura3∆, BUD4-w303, bem1::KanMX6, ABP140-mCherry-NATMX4* | Evolved haploids that grew from spores from yLL66a, and are evolved over 1000 generations. (X indicates the number of evolved generations) |
| 3aA1_X, 3aA2_X, 3aA4_X, 3aA5_X, 3aA6_X, 3aA8_X 3aA10_X | *MATalpha, can1-100, leu2-3,112, his3-11,15, ura3∆, BUD4-w303,* | Evolved ancestor 3a, evolved over 1000 generations. (X indicates the number of evolved generations) |

1. Pan, X., et al., *A robust toolkit for functional profiling of the yeast genome.* Mol Cell, 2004. **16**(3): p. 487-96.
